# Spatiotemporal variations in ischemic heart disease mortality and related risk factors in China between 2010 and 2015: a multilevel analysis

**DOI:** 10.1186/s12889-020-10019-6

**Published:** 2021-01-04

**Authors:** Baohua Wang, Peiyao Li, Fengdie He, Yuting Sha, Xia Wan, Lijun Wang

**Affiliations:** 1 National Center for Chronic and Noncommunicable Disease Control and Prevention, Chinese Center for Disease Control and Prevention, 27 Nanwei Road, Xicheng District, Beijing, 100050 P.R. China; 2 China and Japan Friendship Hospital, Yinghua East Street 2#, Chaoyang District, Beijing, P.R. China; 3 Institute of Basic Medical Sciences Chinese Academy of Medical Sciences, School of Basic Medicine Peking Union Medical College, Beijing, China

**Keywords:** Ischemic heart disease, Risk factors, Multilevel statistical model

## Abstract

**Background:**

To explore the relationship between geographical differences of mortality and related risk factors in ischemic heart disease (IHD) in China.

**Methods:**

Data were collected from the nationally representative China Mortality Surveillance System to calculate annual IHD mortality counts (2010–2015). Descriptive analysis was used to analyze the IHD mortality among Chinese population from 2010 to 2015. Negative binomial regression was used to investigate potential spatiotemporal variation and correlations with age, gender, urbanization, and region.

**Results:**

The overall IHD mortality was 221.17/100,000, accounting for 1.51 million deaths in 2015. The standardized IHD mortality rate increased by 5.51% from 2010 to 2015 among people aged 40 years and older. Multilevel analysis indicated significant differences in gender, regions, and age. High urbanization rate (risk ratio [RR] = 0.728, 95% confidence interval [CI] = (0.631, 0.840)) and average high-density lipoprotein (HDL) (RR = 0.741, 95%CI: 0.616,0.891) were negatively associated with IHD mortality. IHD mortality was significantly higher in populations with a low rate of medical insurance coverage (RR = 1.218, 95%CI: 1.007, 1.473), as well as the average body mass index (BMI) (RR = 1.436, 95%CI: 1.135, 1.817) and systolic blood pressure (SBP) (RR = 1.310, 95%CI: 1.019, 1.684). While the relationship with current smoking rate, excessive intake of red meat, insufficient vegetable or fruits intake didn’t show the statistical significance. The negative correlation between the average sedentary time and IHD mortality was not conclusive due to the possible deviation of the data.

**Conclusions:**

The mortality of IHD showed an upward trend for people aged 40 years and older in China during 2010–2015, which should be paid attention to. Therefore, some risk factors should be controlled, such as SBP, overweight/obesity. HDL is a protective factor, as well as higher urbanization rate, family income level, and medical insurance coverage.

## Background

Ischemic heart disease (IHD) has caused 30% of total annual deaths, which become the leading cause of death and a major economic burden worldwide [1, 2]. Two out of five deaths were due to cardiovascular disease, especially in rural areas [3]. IHD has imposed a heavy burden on families and society overall, and has become a major public health issue.

The multilevel model (MLM), also called linear mixed model or hierarchical linear model, has become increasingly popular in public health for analyzing data with repeated measurements or data organized in nested levels [4]. The advantages of it are as follow: (i) It could effectively estimate the regression coefficient, (ii) provide the confidence interval (CI) and the hypothesis test by using the aggregation information and the correct standard error; (iii) and introduce the co-variable at any level to explore the effects of all variables at any level for researchers [5].

The distribution of IHD in China has spatial and temporal differences. For example, from 1991 to 2000, counties with high IHD mortality were located in northwest, north, and northeast China, while from 2004 to 2009, the high mortality rate areas expanded to the north, central and southern regions. Counties with an increase in IHD mortality of more than 100% are concentrated in the Northeast and South [6]. In 2015, the highest age-standardized mortality rate per 100,000 people of IHD was Heilongjiang Province (187.4, 95% CI: 161.6–217.5), and the lowest was Shanghai (44.2, 95% CI: 37.0–53.1). Geographically, the age-standardized death and DALY rate in the southern provinces were lower than those in the northern provinces, especially in the southeastern coastal provinces [7].

Therefore, the data from Disease Surveillance Systems (DSP), with nationally representative, was used to investigate temporal trends and geographical variations in IHD mortality in China from 2010 to 2015. The key objectives of this study are as follows: (i) to investigate the levels and characteristics of IHD mortality across China from 2010 to 2015, (ii) to explore whether there are geographical differences in IHD mortality rates and to determine whether those differences vary over time, and (iii) to establish a multilevel model for the analysis of factors affecting IHD in the Chinese population.

## Methods

### Data source

The International Classification of Diseases 10 (ICD-10) was used to define IHD mortality in the China Mortality Surveillance System. The relevant codes included I20-I25(Angina pectoris, Acute myocardial infarction, Subsequent myocardial infarction, Chronic ischemic heart disease). More information on DSP system were described elsewhere [8]. In general, it comprises 161 counties or districts across all 31 provinces, municipalities, and autonomous regions in Mainland China, providing coverage of approximately 73 million people [9]. County- and district-level (i.e., DSP) population counts cross- classified by 5-year age group and gender were obtained from the Chinese census in 2000 and 2010. These counts were used as a reference population for calculating the age-standardized mortality rates of IHD and in statistical models. The total population for each DSP in the years 2010–2015 was then estimated assuming an exponential growth across the time period, in line with the methodology used in the estimation of mortality rates in China in the Global Burden of Disease Study (GDB) [10].

In total, 161 DSPs were classified as ‘rural’ (*n* = 97) or ‘urban’ (*n* = 64) according to administrative characteristics. Meanwhile, according to their regional affiliation by the China National Bureau of Statistics [11], all the DSPs were classified into seven regions [12].

In addition, person-level data from the China Chronic Disease Risk Factor Surveillance (CDRFS) in 2013 was used to estimate the prevalence of risk factors at the DSP level. Detailed design and methods were described elsewhere [13]. CDRFS was a cross-sectional population-based survey with national representative, which included 98,658 adult participants from all 161 DSP sites by using a complex, multistage, probability sampling design. A questionnaire including information on demographic characteristics, medical history, and lifestyle factors was conducted by trained interviewers.

### Model information

Based on the characteristics of death data, negative binomial regression was selected to use for over-dispersion of the mortality counts [14]. An initial model included fixed effects for age group, gender, and year. A three-level model was fitted, with the DSP/gender/5-year age group cross-classification at Level 1, county at Level 2, and province at Level 3. The natural logarithm of the equivalently classified denominator counts was fitted as an offset to account for local population distribution. The behavioral risk factor variables and socio-economic indicators are added to the model one by one to calculate the percentage variance change (PCV) of the variance between the province and county levels. The relative risk (RR) and 95% confidence interval (CI) of all fixed effect parameters can be obtained through the model.

In this study, each behavioral risk factor index and socio-economic index were divided into three grades: low, medium, and high according to tertiles. Specific indicators included: urbanization rate, current smoking rate, excessive intake of red meat, insufficient intake of fruits and vegetables, average sedentary time, average body mass index (BMI), average systolic blood pressure (SBP), fasting blood glucose (FBG) average level, high-density lipoprotein (HDL) average level, average total cholesterol level, triglyceride level, non-medical insurance ratio, family income level.

### Statistical analysis

The median rate ratios (MRRs) was calculated by fitting the variance of random effects. MRRs is calculated using the same equation as the median odds ratio (MOR) and has a similar interpretation; that MRRs equal to 1 suggests no geographic variation in the outcome variable, whereas values above 1 indicate the necessity of taking context into account [15]. A common application of multilevel models is to apportion the variance in the response according to the different levels of the data [16]. Much interest is focused on the amount of variation attributable to the high level, because this provides a quantitative estimation of “where the action lies”. Such a measure in simple variance components, continuous response, variance partition coefficient (VPC) is often referred to as the “intra-class correlation, ICC”. In the two-level variance component model, VPC represents the proportion of level 2 (high level) variance to the total variance:

$$ VPC=\frac{\sigma_{u0}^2}{\sigma_e^2+{\sigma}_{u0}^2} $$

where ${\sigma}_{u0}^2$ is the variance related to the level 2 (high level), ${\sigma}_e^2$ is the level1(low level). More statistical notations are referred elsewhere [17, 18].

All fixed effect parameters were exponentiated to RRs and 95% CIs. All statistical analyses were conducted in MLwIN v2.30.

## Results

### IHD mortality in the Chinese population from 2010 to 2015

The IHD crude mortality rates was 221.17 per 100,000 in 2015 and increased by 23% (from 88.16 to 108.74 per 100,000) from 2010 to 2015 in the overall population (Table 1). The age-standardized mortality rates of IHD among people aged 40 years and older increased 5.51% (from 145.95 to 153.99 per 100,000) from 2010 to 2015(Table 2). Gender differences were also observed, with IHD mortality increasing more slowly for males (5.18 per 100,000) than females (9.51 per 100,000). The disparity between urban and rural residence was notable. IHD mortality rates during the study period decreased by 1.53% (from 133.89 to 132.36) in urban areas but increased by 14.88% (from 154.79 to 169.67) in rural areas.

**Table 1:** Crude mortality of IHD in people over 40 years old in different regions from 2010 to 2015 (1/100,000)

	Urban	Rural	Male	Female	East	Central	West	Total
2010	183.04	208.87	212.24	183.60	201.69	227.20	152.40	197.99
2011	185.65	212.93	216.76	187.06	199.25	234.41	157.91	201.71
2012	190.13	235.96	230.59	202.48	206.65	250.34	184.24	216.59
2013	202.31	248.77	244.87	213.87	220.27	263.63	195.52	229.39
2014	194.78	233.20	229.40	203.91	209.10	245.55	189.70	216.67
2015	198.73	236.58	233.56	208.80	215.34	254.34	186.47	221.17

**Table 2:** Standardized mortality of IHD in people over 40 years of age in different regions from 2010 to 2015 (1/100,000)

Year	2010	2011	2012	2013	2014	2015
Urban						
Male	152.97	153.31	154.50	159.38	150.23	149.91
Female	115.12	113.28	115.86	120.07	116.44	114.43
Total	133.89	133.11	135.17	139.83	133.50	132.36
Rural						
Male	183.66	184.82	202.73	212.04	195.63	195.00
Female	128.16	131.13	145.43	152.17	142.18	144.92
Total	154.79	156.88	173.15	181.49	168.30	169.67
Urban+rural						
Male	170.59	171.62	181.87	189.37	175.38	175.77
Female	122.56	123.69	132.81	138.48	130.81	132.07
Total	145.95	147.04	156.93	163.76	152.99	153.99
East						
Male	165.17	160.66	166.15	171.62	161.97	164.21
Female	118.50	116.91	120.17	125.53	117.53	117.93
Total	140.93	138.08	142.53	148.34	139.42	140.83
Central						
Male	202.93	206.87	216.34	224.24	204.83	208.63
Female	144.46	148.01	158.15	165.01	155.29	159.37
Total	173.01	176.67	186.84	194.36	179.93	184.09
West						
Male	135.27	139.28	158.79	169.69	158.3	151.91
Female	97.41	99.11	117.05	121.42	120.61	118.93
Total	116.05	118.84	137.76	145.60	139.62	135.71

**Fig. 1:**
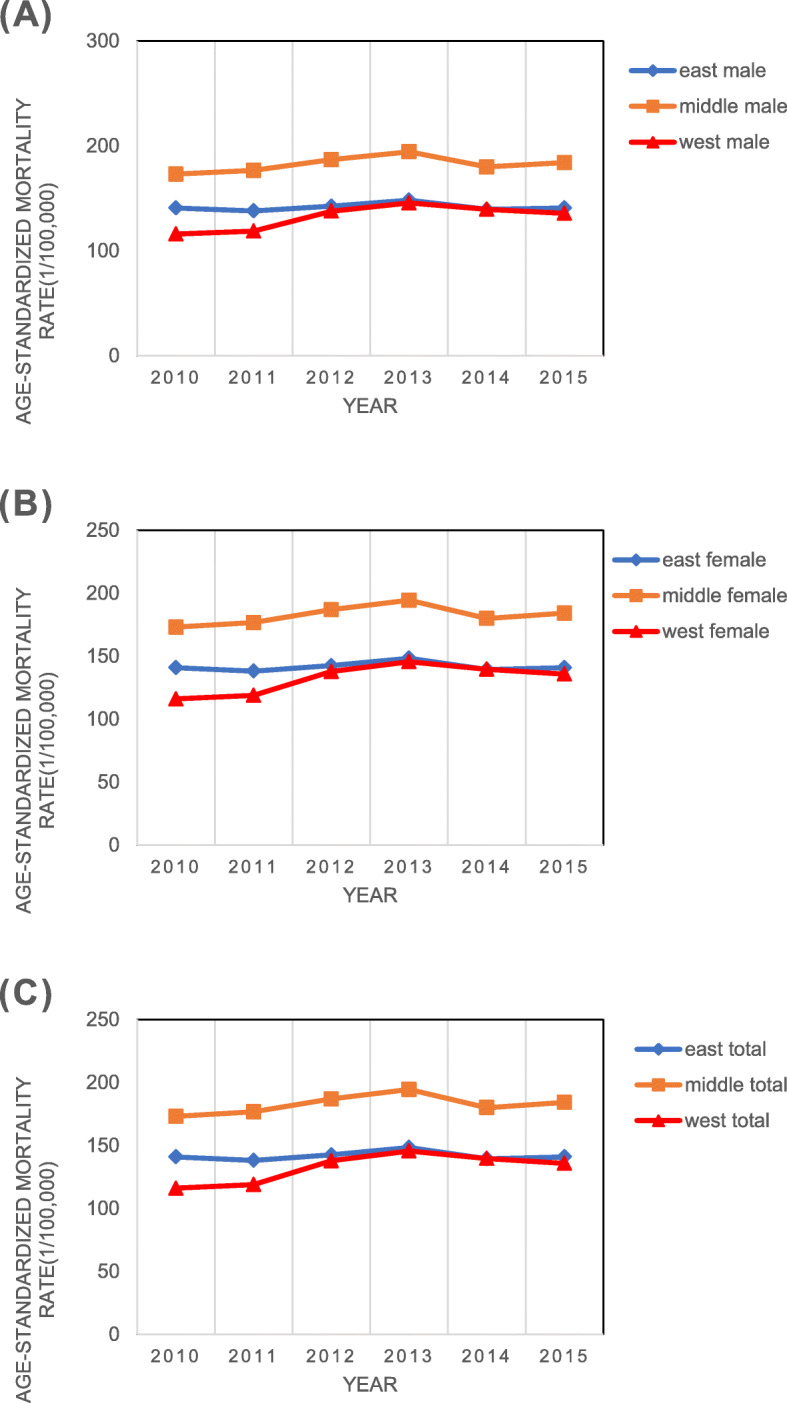
Age-standardized mortality rates of IHD in people over 40 years of age in different regions from 2010 to 2015 (1/100,000)

The mortality rate was apparently different between males and females, and between urban and rural regions during the study period (Fig. 1). Age-standardized IHD mortality rates in females and urban areas were lower than in males and rural areas. In this study, the superposition effect of gender and urban/rural areas in age-standardized mortality rates showed that the IHD mortality rates were highest in rural males and were lowest in urban females. In urban areas, the age-standardized IHD mortality rates decreased 2.00% for males (from 152.97/ 100,000 in 2010 to 149.91/ 100,000 in 2015), and 0.6% for females (from 115.12/100,000 in 2010 to 114.43/ 100,000 in 2015), respectively. However, in rural areas, the age-standardized IHD mortality rates increased 6.17% for males (from 183.66/ 100,000 in 2010 to 195.00/ 100,000 in 2015) and 13.8% for females (from 128.16/100,000 in 2010 to 144.92/ 100,000 in 2015), respectively (see Fig. 1a, b).

As shown in Table 2, mortality rates were highest in the central region and lowest in the west. Mortality rates in the eastern region primitively increased and then decreased in total (from 140.93/100,000 to 140.83/100,000). Nevertheless, mortality rates in the western and central region increased over time (from 116.05/100,000 to 135.71/100,000, from 173.01/100,000 to 184.09/100,000, respectively). The increasing slope was highest from 2010 to 2013 (Fig. 1c).

Gender and region stratified analyses showed the mortality rates were different. For males, the mortality rates in the eastern region showed a downward trend (from 165.17/100,000 to 164.21/100,000). However, the age-standardized IHD mortality rates increased by 6.40% from 173.01/100,000 to 184.09/100,000 in the central region, and it increased by 16.94% from 116.05/100,000 to 135.71/100,000 in the western region. For females, in total, the age-standardized IHD mortality rates slightly decreased from 118.50/100,000 to 117.93/100,000 in the eastern region. The mortality of western and central regions increased in total (from 97.41/100,000 to 118.93/100,000, from 144.46/100,000 to 159.37/100,000, respectively), while the mortality rates were lower after 2013 (Fig. 1).

### IHD mortality in the Chinese population from 2010 to 2015 in multilevel models

Multilevel model 1 (Table 3) indicates that age and the gender-adjusted IHD mortality increased between 2010 and 2015 (RR = 1.016, 95% CI: 1.012, 1.020), and it increased with age (RR = 1.839, 95% CI: 1.835, 1.842). IHD mortality was lower among females (RR = 0.622, 95% CI: 0.612, 0.632). The provincial and DSP intercept variance was statistically significant (MRRp = 1.514 and MRRd = 1.459), which indicated the necessity of using multilevel models. IHD mortality rate was explained by adjustment for region in model 2. Compared to the northern region, IHD mortality rate was significantly lower in the east (RR = 0.435, 95% CI: 0.307, 0.616), southwest (RR = 0.360, 95% CI: 0.246, 0.525), and south (RR = 0.512, 95% CI: 0.329, 0.795). The provincial and DSP intercept variance was significantly different (MRRp = 1.258 and MRRd = 1.430). To explore the role of socioeconomic factors on IHD mortality, the urbanization rate of each DSP was added in multilevel model 3. Comparing with low urbanization rate areas, the RR and 95% CI of the areas with a high urbanization rate was 0.728(0.631, 0.840).

**Table 3:** Multilevel analysis of spatiotemporal changes in mortality of ischemic heart disease in China from 2010 to 2015

	Model 1	Model 2	Model 3	Model 4	Model 5	Model 6
Fixed effect	RR (95%CI)					
Year	1.016 (1.012,1.020)*	1.020 (1.016,1.024)*	1.019 (1.015,1.023)*	1.019 (1.015,1.023)*	1.019 (1.015,1.023)*	1.019 (1.015,1.023)*
Gender (ref:male)	1	1	1	1	1	1
Female	0.622 (0.612,0.632)*	0.618 (0.609,0.628)*	0.618 (0.608,0.627)*	0.618 (0.608,0.627)*	0.619 (0.609,0.629)*	0.619 (0.610,0.629)*
Age (5 years old as an age group)	1.839 (1.835,1.842)*	1.848 (1.844,1.851)*	1.850 (1.846,1.853)*	1.850 (1.846,1.853)*	1.852 (1.848,1.855)*	1.852 (1.848,1.855)*
Area (ref: north)		1	1	1	1	1
East		0.435 (0.307,0.616)*	0.442 (0.317,0.615)*	0.484 (0.346,0.677)*	0.598 (0.446,0.800)*	0.641 (0.487,0.843)*
Central		0.720 (0.475,1.094)	0.705 (0.475,1.048)	0.710 (0.484,1.043)	0.837 (0.604,1.159)	0.845 (0.629,1.137)
North		0.512 (0.329,0.795)*	0.531 (0.350,0.805)*	0.572 (0.376,0.870)*	0.739 (0.509,1.073)	0.743 (0.527,1.047)
Weatsouth		0.360 (0.246,0.525)*	0.334 (0.233,0.478)*	0.361 (0.250,0.520)*	0.472 (0.340,0.656)	0.509 (0.375,0.691)*
Westnorth		0.865 (0.589,1.270)	0.824 (0.574,1.185)	0.757 (0.526,1.090)	0.770 (0.564,1.052)	0.795 (0.596,1.060)
Eastnorth		1.051 (0.688,1.605)	1.051 (0.705,1.568)	0.994 (0.668,1.480)	1.023 (0.754,1.389)	0.948 (0.717,1.255)
Urbanization rate (ref:low)			1	1	1	1
Middle			0.949 (0.816,1.104)	0.987 (0.842,1.157)	1.043 (0.897,1.213)	1.002 (0.863,1.163)
High			0.728 (0.631,0.840)*	0.771 (0.646,0.920)*	0.820 (0.684,0.984)	0.819 (0.672,0.998)*
Current smoking rate (ref: low)				1	1	1
Middle				0.971 (0.826,1.143)	0.952 (0.820,1.105)	0.952 (0.824,1.101)
High				1.134 (0.947,1.358)	1.133 (0.954,1.346)	1.082 (0.914,1.281)
Excessive red meat intake (ref: low)				1	1	1
Middle				0.890 (0.757,1.048)	0.893 (0.765,1.043)	0.890 (0.766,1.032)
High				0.891 (0.733,1.084)	0.918 (0.763,1.103)	0.900 (0.756,1.072)
Insufficient Vegetable and fruit intake (ref: low)				1	1	1
Middle				0.943 (0.811,1.096)	0.941 (0.811,1.092)	0.927 (0.802,1.071)
High				0.901 (0.767,1.058)	0.898 (0.769,1.048)	0.884 (0.762,1.026)
Average meditation time (ref: low)				1	1	1
Middle				0.895 (0.767,1.045)	0.883 (0.763,1.023)	0.851 (0.739,0.980)
High				0.933 (0.789,1.105)	0.869 (0.737,1.025)	0.868 (0.741,1.018)
Average BMI (ref:low)					1	1
Middle					1.135 (0.939,1.373)	1.050 (0.870,1.268)
High					1.495 (1.172,1.906)*	1.436 (1.135,1.817)*
Average SBP (ref:low)					1	1
Middle					1.150 (0.983,1.346)	1.149 (0.986,1.339)
High					1.294 (1.007,1.663)*	1.310 (1.019,1.684)*
Average FBG (ref:low)					1	1
Middle					1.069 (0.907,1.261)	1.061 (0.905,1.243)
High					1.093 (0.909,1.314)	1.079 (0.903,1.290)
Average HDL (ref:low)					1	1
Middle					0.830 (0.706,0.977)	0.816 (0.692,0.962)*
High					0.746 (0.619,0.899)	0.741 (0.616,0.891)*
Average total cholesterol (ref:low)					1	1
Middle					0.932 (0.772,1.125)	0.899 (0.747,1.080)
High					1.062 (0.838,1.346)	1.046 (0.828,1.321)
Triglyceride level (ref:low)					1	1
Middle					1.012 (0.857,1.196)	1.080 (0.913,1.278)
High					1.081 (0.897,1.302)	1.079 (0.899,1.295)
No health insurance ratio (ref:low)						1
Middle						1.137 (0.964,1.340)
High						1.218 (1.007,1.473)*
Household income level (ref:low)						1
Middle						1.146 (0.989,1.327)
High						0.886 (0.741,1.059)
Random effect						
Variance (SE) Provincial level	0.189 (0.057)	0.058 (0.023)	0.054 (0.020)	0.049 (0.019)	0.022 (0.011)	0.015 (0.009)
MRR	1.514	1.258	1.248	1.235	1.152	1.124
PCV	–	70.1%	72.2%	74.9%	89.8%	94.7%
Variance (SE) Monitoring point level	0.157 (0.020)	0.141 (0.018)	0.114 (0.015)	0.112 (0.015)	0.095 (0.012)	0.089 (0.011)
MRR	1.459	1.430	1.380	1.376	1.342	1.329
PCV	–	10.4%	29.4%	30.1%	35.6%	38.1%
VPC	–	0.145	0.183	0.161	0.051	0.028

* *p* < 0.05; *MRR* Median rate ratio, *PCV* Proportional change in variance in Model x compared to Model 1, *VPC* Variance partition coefficient, *DSP* Disease surveillance points

Lifestyle factors (smoking, red meat intake, vegetable and fruit deficiency, sedentary time) were included in the multilevel model 4. The results showed that smoking, red meat intake, vegetable and fruit deficiency, and sedentary time did not have statistically significant. Compared to model 3, model 4 increased approximately 0.7% of the DSP-level PCV in IHD mortality. In model 5, which incorporated the six metabolic risk factors (average BMI, SBP, mean FBG, HDL cholesterol, cholesterol level and triglyceride level), only average BMI and SBP were positively associated with IHD mortality (RR = 1.495, 95% CI: 1.172, 1.906, RR = 1.294, 95% CI: 1.007, 1.663). Moderate and high HDL cholesterol levels were negatively correlated with IHD mortality (RR = 0.830, 95%CI:0.706, 0.977, RR = 0.746, 95%CI: 0.619,0.899, respectively). In order to know the role of personal economic situation on the IHD mortality, health insurance rate and family income level were added into multilevel model 6. A low health insurance rate was positively associated with the IHD mortality (RR = 1.218, 95% CI: 1.007,1.473), indicating the importance of health insurance coverage expansion. The relationship between family income level and IHD mortality is not obvious (RR = 0.886, 95% CI:0.741,1.059). Moderate sedentary time was negatively correlated with IHD mortality (RR = 0.851, 95% CI: 0.739,0.980) in model 6, but it showed no correlation with IHD mortality in model 4 and 5. In model 6, approximately 94.7 and 38.1% of the provincial-level and the DSP-level variation, respectively, in IHD mortality was explained by considering behavioral risk factors and socioeconomic factors.

The VPC was reduced when lifestyle variances were introduced in the model, which suggested that some part of the contextual phenomenon related to IHD on monitoring point was attributable to provincial deprivation.

## Discussion

Cardiovascular and cerebrovascular diseases are the leading cause of death worldwide. The results of this study showed that the IHD mortality rate was 108.74/100,000 in 2015, accounting for 39.74% of cardio-cerebrovascular disease. IHD mortality in the males was higher than that in the females. For people aged 40 years and older, the crude IHD mortality rate was 221.17/100,000 in 2015. Furthermore, the results of our analysis showed that the crude IHD mortality rate was higher in the rural males and in the central region, compared to urban females in other regions. This might be a result of stress and lifestyle. For example, males tend to have bad habits such as drinking [19] and smoking [20]. Regional differences might be related to several factors, such as the level of socioeconomic status, availability and affordability of health services, consciousness of prevention, urbanization, economic growth, and epidemiological transition on cardiovascular health [21, 22].

Our results show that the crude IHD mortality rate increased by 23.34% during the 2010–2015. After eliminating the influence of the aging population, the standardized IHD mortality rate showed an upward trend. The age-standardized IHD mortality among people aged 40 years and older also increased from 2010 to 2015 [10].

Many large prospective cohort studies have shown that hypertension, hyperlipidemia, diabetes, smoking, and obesity are the main risk factors for IHD [23]. Elevated blood pressure is the most important determinant risk factors for stroke [24]. Unhealthy lifestyles such as smoking, drinking, high salt diet, dietary imbalance, and lack of physical activity can directly or indirectly affect the incidence and mortality rate of IHD [25]. In this study, a multilevel model was used to study the relationship between the regional and the other related risk factors on the IHD mortality rate.

Study have found that mortality from IHD is associated with socioeconomic status (low education, poverty, working and living conditions). Developed regions, with greater healthcare financing and efficient primary health care, have lower mortality rates [26]. The results of our multilevel model showed that a high urbanization rate was negatively correlated with the IHD mortality rate; and the higher the urbanization rate, the lower IHD the mortality rate. The rapid development of China’s economy, great changes have taken place in people’s diet, especially in the urban areas. They eat more meat and milk, high calorie foods, foods high in fat and protein, and less whole grains and vegetables. These lifestyle changes increase the risk of IHD, which is consistent with results from individual-level prospective studies [27]. However, moderate intake of fruits, vegetables, and beans can reduce the risk of death from cardiovascular disease [28]. The level of medical treatment in urban regions, the health infrastructure, institutional care, palliative care, and hospice care have been significantly improved. The results of this article also support that the conclusion that the IHD mortality rate is basically stable in the urban and eastern regions, while rising in rural and western areas.

Many studies have confirmed that the risk factors of metabolism are closely related to the pathogenesis of cardio-cerebrovascular diseases. The prominent risk factors are hypertension, hyperlipidemia, diabetes, overweight/obesity [29]. Our study showed that high BMI level and high SBP level were positively correlated with IHD, with RRs of 1.495 and 1.294, respectively. Moderate HDL level and high HDL level were negatively correlated with IHD, with RRs of 0.830 and 0.746, respectively.

We found that BMI was positively correlated with IHD mortality, which is consistent with the prospective study [30]. A cohort study in South Korea showed that SBP > 120 mmHg and DBP > 70 mmHg had a trend of having increased CVD incidences [31]. However, there is no statistical significance for the relationship between the prevalence of hypertension and IHD mortality. This might be related to the diagnostic basis of hypertension. Many studies have shown that pulse pressure is an independent factor for cardio-cerebrovascular diseases. HDL is also associated with IHD mortality. A comparison of the average HDL-C levels between Korean and American populations was conducted in order to estimate the optimal HDL-C threshold for predicting cerebrovascular accidents and IHD [32].

In recent years, as the prevalence of IHD increased, the medical expenses of IHD has grown. Because of the high cost of treating IHD in hospital, an increase in average family income will be beneficial to the reduction of IHD mortality. The results of our study support the conclusion that the IHD mortality rate was significantly higher in those without adequate medical insurance (RR 1.218, 95% CI: 1.007, 1.473). Thus, expanding the proportion of those with health insurance benefits will decrease IHD mortality.

In addition to metabolic factors, smoking, drinking, inadequate physical activity, unhealthy diet and other behavioral factors have been proved to have an impact on the incidence of IHD and its mortality rate in many studies. We found that smoking, red meat intake, and insufficient ingestion of vegetables and fruits did not have a significant effect on IHD mortality rate. In the analysis of the reasons, on the one hand, when we adopt the multilevel analysis, the behavioral factors are in the level of the monitoring point, which does not reflect the specific situation of the case. Furthermore, quality control measures were not consistent, leading to uneven data and recall bias. To ensure the accuracy of our data, we provided routine training to coding staff and had a regular checking and verification process to avoid coding errors.

In addition, our study found that the average sedentary time had a negative correlation with IHD mortality among people aged 40 years and over. A cohort study abroad also found that difference in risk of IHD was not observed between sedentary and non-sedentary employees [33]. However, a Canadian study [34] found that sedentary lifestyles were associated with cardiovascular disease and the incidence and mortality of cancers, especially type-2 diabetes. Zhou et al. [35] studied the relationship between sedentary time and cardio-cerebrovascular disease in Beijing, and they found that sedentary lifestyle was significantly associated with fasting blood glucose, triglycerides, cholesterol, and LDL.

An important strength of this study is that the data on IHD in this article comes from the long-term monitoring data of 161 DSPs across the country. It is a nationally representative national death cause monitoring network data, which ensures the reliability, accuracy and continuity of the death cause data. Additionally, the data spans from 2010 to 2010, it covered the whole country and provided important data for studying the temporal and spatial changes of IHD and the differences between urban and rural areas and gender. Another important strength is that the main risk factor monitoring data comes from the 2013 China Chronic Disease and Risk Factor Monitoring. This monitoring covers 302 monitored counties and districts in 31 provinces (autonomous regions and municipalities). The survey results are both nationally representative and provincial-level representatives. Therefore, from the perspective of data sources, this article uses data from large-scale national surveillance networks, including data on the cause of death, behavioral risk factors, etc., to comprehensively study the relationship between the temporal and spatial changes of IHD and risk factors.

There are limitations in this study. IHD often has many complications, and the chain of disease causes of death is complex and diverse, which makes the judgment of the fundamental cause of death complicated and difficult. At the same time, China has a vast land and abundant resources, the level of economic development is quite different, and a large number of deaths occur at home, which might cause deviations in the judgment of the fundamental cause of death. Therefore, the death data of IHD in this article might be underestimated and misreported. In addition, the quality control of each monitoring point is different, and the quality of the data is different. These potential factors might affect the results of the study to a certain extent.

## Conclusion

Generally, IHD mortality rate increased during 2010–2015. IHD mortality in the population aged 40 years and over showed significantly differences between gender, urban/rural areas, and regions. The high level of SBP, overweight / obesity (BMI) were risk factors for IHD, while high density lipoprotein (HDL) was a protective factor. Immediate action via primary and secondary preventions to enhance the prevention and management of IHD are required specifically in China. Urbanization rate, medical insurance coverage, and family income were conducive to the reduction in IHD mortality. Measures to further develop economy, improve people’s living standards, continue to promote urbanization, increase investment in the medical field, and expand medical insurance coverage were recommended to tackle this crucial health inequality.

## Data Availability

The datasets used and/or analysed during the current study are available from the corresponding author on reasonable request.

## Abbreviations

IHD: Ischemic Heart Disease
RR: Risk Ratio
CI: Confidence Interval
BMI: Body Mass Index
SBP: Systolic Blood Pressure
HDL: High Density Lipoprotein
CVD: Cardiovascular Diseases
CHD: Coronary Heart Disease
DALY: Disability Adjusted Life Year
ICD-10: International Classification of Disease 10
DSP: Disease Surveillance Point
GBD: Global Burden Disease
CDRFS: Chronic Disease Risk Factor Surveillance
FBG: Fasting Blood Glucose
MRRs: Median Rate Ratios
PCV: Percentage Change Variance
MOR: Median Odds Ratio
VPC: Variance Partition Coefficient
ICC: Intra-Class Correlation
